# Trimetazidine Increases Plasma MicroRNA-24 and MicroRNA-126 Levels and Improves Dyslipidemia, Inflammation and Hypotension in Diabetic Rats

**DOI:** 10.22037/ijpr.2020.1101144

**Published:** 2020

**Authors:** Fatemeh Ramezani-Aliakbari, Mohammad Badavi, Mahin Dianat, Seyyed Ali Mard, Akram Ahangarpour

**Affiliations:** a *Department of Physiology, School of Medicine, Hamadan University of Medical Sciences, Hamadan, Iran.*; b * Department of Physiology, School of Medicine, Ahvaz Jundishapur University of Medical Sciences, Ahvaz, Iran. *; c *Persian Gulf Physiology Research Center, School of Medicine, Ahvaz Jundishapur University of Medical Sciences, Ahvaz, Iran.*; d *Atherosclerosis Research Center, Ahvaz Jundishapur University of Medical Sciences, Ahvaz, Iran. *; e *Diabetes Research Center, Ahvaz Jundishapur University of Medical Sciences, Ahvaz, Iran.*

**Keywords:** Diabetes, Endothelium, Inflammation, MicroRNAs, Oxidative Stress, Trimetazidine

## Abstract

Trimetazidine (TMZ) improves endothelial dysfunction. However, its beneficial effect on endothelial miRNAs is unexplored in diabetes. The aim of the present study was to evaluate the effects of TMZ on plasma miRNA-24 and miRNA-126, dyslipidemia, inflammation, and blood pressure in the diabetic rats. Adult male Sprague-Dawley rats were randomly assigned into four groups (250 ± 20 g, n = 8): a control (C), an untreated diabetic (D), a diabetic group administrated with TMZ at 10 mg/kg (T10), and a diabetic group administrated with TMZ at 30 mg/kg (T30) for eight weeks. Diabetes was induced by injection of alloxan (120 mg/kg). The plasma levels of miR-24, miR-126, lipid profile, malondialdehyde (MDA), tumor necrosis factor-alpha (TNF-α) and interleukin-6 (IL-6), blood glucose, body weight and systolic blood pressure were measured. The diabetic rats showed decreased plasma miR-24, HDL-c (*P* < 0.05), miR-126 (*P* < 0.01), body weight changes percent, body weight, and systolic blood pressure (*P *< 0.001) and increased triglycerides (TG), VLDL-c (*P *< 0.05), TNF-α, total cholesterol (TC) (*P *< 0.01) glucose, MDA and IL-6 (*P *< 0.001). Interestingly, all these changes were significantly improved by TMZ treatment. Our findings propose that TMZ has protective effects on decreased plasma miR-24 and miR-126 levels, inflammation, dyslipidemia and hypotension, and it may participate in endothelial dysfunction and atherosclerosis.

## Introduction

Diabetes Mellitus is a metabolic disorder that induces many complications with an increasing frequency world- wide (1). Diabetes is associated with hyperglycemia, hyperlipidemia, increased oxidative stress, and cardiovascular disorders (2). Micro- and macro-vascular complications induced by diabetes are the causes of morbidity and mortality in diabetic patients (3). Under increased oxidative stress conditions including diabetes, free radical generation is elevated and induces endothelial dysfunction and vascular disorder (4). Increased inflammatory cytokines, leukocyte adhesion, endothelial cell apoptosis, and vascular permeability have been observed in diabetes (5, 6).

MicroRNAs (miRNAs) are small (22-25 nucleotides), single-stranded, non-protein encoding RNAs that are involved in target gene expression through translation and degradation (7). MiRNAs have been demonstrated to be important modulators of biological processes including development, differentiation and metabolism (8), which have been regarded as new and non-invasive markers in all body fluids, including plasma, urine, saliva, tears, breast milk, and cerebro-spinal fluid (9). In addition, previous studies have shown that miRNAs can play an important role in the pathogenesis of diabetes and vascular disorders (10-12). MiR-126 as an endothelial-enriched miRNA has been implicated in angiogenesis, integrity, and vascular system regeneration, suggesting a common angiomiR (12,13). Furthermore, increasing studies have demonstrated the anti- apoptotic, anti-proliferative and anti-migratory effects of miR-24 on endothelial cells and vascular smooth muscle cells (VSMCs) under ischemia and high glucose conditions (14, 15).

Trimetazidine (TMZ) is a first class anti-anginal drug. The beneficial effects of TMZ on angina pectoris and coronary artery disorders by shifting energy metabolism from fatty acid oxidation to glucose oxidation, reducing the long-chain 3-ketoacyl coenzyme A thiolase, cellular acidosis and increasing ATP production in the heart and vascular dilation have been reported (16-18). Furthermore, previous studies have indicated that TMZ can regulate the permeability of mitochondrial transition pore causing ischemia-reperfusion injury and signaling damage in the heart (19-22). Both clinical and experimental studies on cardiovascular responses in TMZ treatment have revealed reduction of malondialdehyde (MDA), lipid hydro-peroxidase levels and improvement of endothelial dysfunction, right ventricular function and fibrosis by antioxidant effect and increasing miR-21 expression (1,23,19). Taken together, these findings from related studies make potent evidence that TMZ has beneficial effects on cardiovascular system. However, the role of TMZ in plasma miR-24 and miR-126 levels improvement in diabetes was still unknown. Therefore, the present study was undertaken to evaluate the effects of TMZ on plasma miR-24 and miR-126 levels, inflammation and hypotension in the diabetic rats.

## Experimental

*Drugs*

Trimetazidine (1-​(2,3,4-​TRIMETHOXYBENZYL)​ PIPERAZINE DIHYDROCHLORIDE) and Alloxan monohydrate were obtained from Sigma Chemical Co. (St. Louis, MO, U.S.A.) and also Ketamine (10%) and Xylazine (2%) were purchased from Alfasan Co (Woderen- Holland).

*Experimental groups*

Adult male Sprague-Dawley rats weighing 250 ± 20 g were purchased from Ahvaz Jundishapur University of Medical Sciences Animal House Center, Ahwaz, Iran. The animals were housed under standard conditions (temperature 22 ± 2 °C, 12 h light/dark cycle) and free access to food and water. All the animal protocols were approved by the Institutional Animal Care and Ethics Committee of the Ahvaz Jundishapur University of Medical Sciences and were conducted in accordance with the guidelines for the care and use of laboratory animals (grant No. APRC-94-25). After a week, the animals were randomly divided into four groups (n = 8): control (C), diabetic (D) and diabetic treated with TMZ at 10 mg/kg (T10) and at 30 mg/kg (T30) (24). TMZ was dissolved in normal saline and administrated by gavage once daily for 8 weeks. The rats in the C and D groups were gavaged with saline only. The animals in the high-dose (30 mg/kg) TMZ treated group were given 3 mL/kg/day TMZ and in low dose (10 mg/kg) TMZ treated group were given 1 mL/kg/day TMZ. The control and untreated diabetic groups were given 3 mL/kg/day normal saline.

*Induction of diabetes*

Diabetes was induced by a single intraperitoneal injection of alloxan in the animals (120 mg/kg). Six h later, the rats were given 10% glucose solution for the next 24 h. After 4 days, the alloxan-injected rats that indicated the blood glucose level more than 250 mg/dL, were regarded as diabetic animals. The animals were anesthetized using ketamine and xylazine (80 and 10 mg/kg, respectively). At the end of the experiment, the blood samples were collected from abdominal vein of anesthetized rats and centrifuged at 4000 g for 10 min; the plasma was stored at -80 °C for later assessments, including MDA, TNF-α, IL-6, lipid profile, and molecular measurements.

*Plasma lipid profile, MDA, TNF-a and IL-6*

Total cholesterol (TC), triglycerides (TG), high-density lipoprotein cholesterol (HDL-c), and low-density lipoprotein cholesterol (LDL-c) were measured by commercial kits (Pars Azmune, Tehran, Iran). Moreover, the very low-density lipoprotein cholesterol (VLDL-c) was computed using the following formula: VLDL-c = Total serum triglycerides/5. The plasma MDA and IL-6 levels were evaluated using commercial assay kits (ZellBio, Ulm, Germany). The TNF-α level was measured using appropriate ELISA kit (Diaclone, Besancon, France). The measurements were performed according to the manufacturer’s protocols.

*MiR-24 and miR-126 measurement*

MiRNAs were harvested from 250 µL plasma by Trizol reagent (Qiagen, USA) according to the manufacturer’s protocol and quickly kept at -80 °C (25). RNA concentration was detected using Nanodrop (Nanodrop thermo scientific S.N:D015). Reverse transcription into cDNA was carried out using TaqMan miRNA reverse transcription kit (Qiagen, USA). Real time PCR was performed using Light Cycler® 96 real time PCR system through these conditions: 95 °C for 15 min, then 45 cycles at 94 °C for 15 sec, 55 °C for 30 sec and 70 °C for 30 sec. The miRNAs were augmented by a universal primer and miRNAs primers (Qiagen, USA) (26). In addition, RNase-free water was used as negative control in each step. U6 (MS00033740) was regarded as internal control for the normalization of the miR-24 (MS00005537) and miR-126 (MS00000329) templates. The threshold cycle (Ct) was organized in the exponential phase for each target PCR and acquired from amplification of all miRNAs. The relative expression of miR-24 and miR-126 was analyzed by the 2-(DDCt) method (27).

*Systolic blood pressure*

The systolic blood pressure was measured using tail plethysmography coupled to a recorder system (Powerlab, ADInstruments, Australia) and a computer. The systolic blood pressure was measured 3-4 times, and the mean was regarded as the rat systolic blood pressure.

*Statistical analysis*

The results were presented as mean ± SEM. Kolmogorov-Smirnov test was performed; then, the findings were analyzed using one way analysis of variance (ANOVA) and LSD *post hoc* test or pair *t*-test as appropriate. *P*<0.05 was statistically considered significant.

## Results

*Body weight changes percent, body weight and blood glucose level*

**Figure 1:**
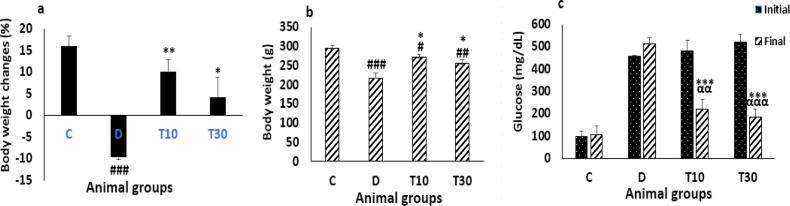
Body weight changes percent (a), body weight (b) and blood glucose level (c) (mean ± SEM, n = 8) in control (C), diabetic (D), diabetic treated with TMZ at 10 and 30 mg/kg (T10 and T30, respectively). ^αα^*P *< 0.01, ^ααα^*P *< 0.001 final *vs*. initial. ^#^*P *< 0.05, ^##^*P *< 0.01, ^###^*P *< 0.001 *vs*. final control group. ^*^P<0.05, ^**^P<0.01, ^***^P < 0.001 *vs*. untreated diabetic group, one-way ANOVA followed by LSD *post hoc* test

Body weight changes percent and body weight were significantly reduced in the diabetic rats compared with the control group (-9.7 ± -0.57 percent *vs*. 16.01 ± 2.46 percent and 218.12 ± 13.15 g *vs. *294.12 ± 8.38 g *P *< 0.001). However, treatment with TMZ at 10 and 30 mg/kg for eight weeks in the diabetic rats significantly inhibited body weight changes percent and body weight reductions compared with the untreated diabetic animals (10.05 ± 2.84 percent and 4.24 ± 4.63 percent *vs*. -9.7 ± -0.57 percent, and 71.42 ± 8.6 g and 255.86 ± 9.2 g *vs*. 218.12 ± 13.15 g, *P *< 0.01 and *P *< 0.05, respectively, Figure 1a, b). 

At the end of the experiment, the plasma glucose did not decrease in the diabetic group. However, the plasma glucose level was significantly improved by TMZ administration at 10 and 30 mg/kg in the diabetic rats compared with the glucose level of the untreated diabetic animals (222.3 ± 41.8 mg/dL and 186 ± 32.4 mg/dL *vs*. 514 ± 27.7 mg/dL, *P *< 0.001 respectively) and with their own initial blood glucose levels (222.3 ± 41.8 mg/dL and 186 ± 32.4 mg/dL *vs.* 479.7 ± 48.3 mg/dL and 520 ± 36 mg/dL, *P *< 0.01 and *P *< 0.001, respectively (Figure 1c).

*Plasma lipid profile*

As shown in Table 1 the diabetic rats showed increased TC (*P *< 0.01), TG and VLDL-c (*P *< 0.05), and decreased HDL-c (*P *< 0.05) when compared with the control group. However, all these changes were significantly improved by TMZ administration at 10 and 30 mg/kg compared with the untreated diabetic group (*P *< 0.01).

*Plasma MDA level*

**Figure 2:**
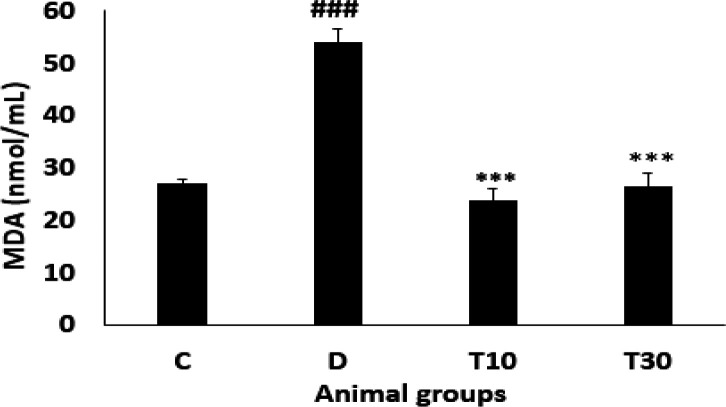
Plasma MDA level (mean ± SEM, n = 8) in control (C), diabetic (D), diabetic treated with TMZ at 10 and 30 mg/kg (T10 and T30, respectively). ^###^*P *< 0.001 *vs*. control group, ^***^P < 0.01 *vs.* untreated diabetic group, one-way ANOVA followed by LSD *post hoc* test

As indicated in Figure 2 plasma MDA level was significantly enhanced in the diabetic group compared with the control rats (54.07 ± 2.6 nmol/mL *vs*. 27.06 ± 0.7 nmol/mL, *P* < 0.001). However, oral treatment with TMZ at 10 and 30 mg/kg for eight weeks in the diabetic animals resulted in a significant reduction of MDA level when compared with the untreated diabetic rats (23.85 ± 2.2 nmol/mL and 26.4 ± 2.6 nmol/mL *vs*. 54.07 ± 2.6 nmol/mL, respectively; *P *< 0.001).

*Plasma TNF-α and IL-6 levels*

**Figure 3:**
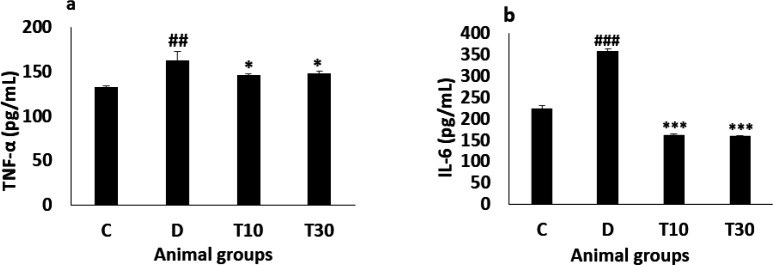
Plasma TNF-α (a) and IL-6 (b) values (mean ± SEM, n = 8) in control (C), diabetic (D), diabetic treated with TMZ at 10 and 30 mg/kg (T10 and T30, respectively). ^##^*P *< 0.01, ^###^*P *< 0.001, vs. control group, ^*^P < 0.05, ^***^P < 0.001 *vs*. untreated diabetic group, one-way ANOVA followed by LSD *post hoc* test

The diabetic animals significantly showed increased TNF-α level compared with the control group (162.12 ± 10.5 pg/mL *vs*. 132.5 ± 1.82 pg/mL, *P *< 0.01) and treatment with TMZ at 10 and 30 mg/kg significantly attenuated this alteration in the diabetic animals compared with the untreated diabetic group (146 ± 1.34 pg/mL and 148 ± 3 pg/mL *vs*. 162.12 ± 10.5 pg/mL, respectively, *P *< 0.05, Figure 3a). Furthermore, IL-6 level in the diabetic rats was significantly more than the control group (357.5 ± 6.8 pg/mL *vs*. 223.27 ± 7 pg/mL, *P *< 0.001), the increased IL-6 level was significantly improved in the diabetic animals treated with TMZ at 10 and 30 mg/kg (161.6 ± 2.6 pg/mL and 159.5 ± 2.2 pg/mL *vs*. 357.5 ± 6.8 pg/mL, respectively; *P *< 0.001, Figure 3b).

*Plasma miR-24 and miR-126 Levels*

**Figure 4:**
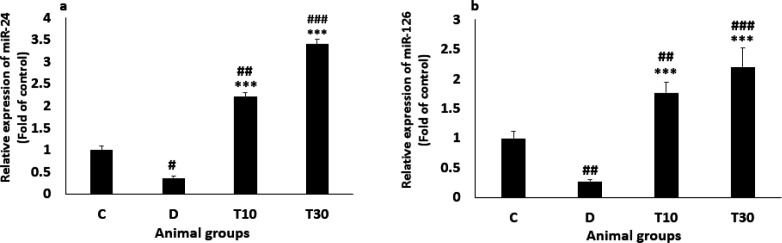
Plasma miR-24 (a) and miR-126 (b) values (mean ± SEM, n = 8) in control (C), diabetic (D), diabetic treated with TMZ at 10 and 30 mg/kg (T10 and T30, respectively). ^#^*P *< 0.05, ^##^*P *< 0.01, ^###^*P *< 0.001, *vs*. control group, ***P < 0.001 *vs*. untreated diabetic group, one-way ANOVA followed by LSD *post hoc* test

The miR-24 level was significantly reduced in the diabetic rats compared with the control group (0.37 ± 0.04 *vs*. 1, *P *< 0.05) but normalized in T10 and T30 groups, suggesting that TMZ treatment significantly increased miR-24 level. (2.21 ± 0.1 and 3.4 ± 0.11 *vs*. 0.37 ± 0.04 respectively, *P *< 0.001, Figure 4a). In addition, the diabetic rats significantly presented decreased miR-126 level compared with the control group (0.27 ± 0.07 vs. 1, *P *< 0.01). Nevertheless, the decreased level of miR-126 was significantly inhibited by treatment with TMZ at 10 and 30 mg/kg (1.77 ± 0.18 and 2.2 ± 0.33 *vs*. 0.27 ± 0.07, respectively; *P *< 0.001; Figure 4b). 

*Systolic blood pressure*

**Figure 5:**
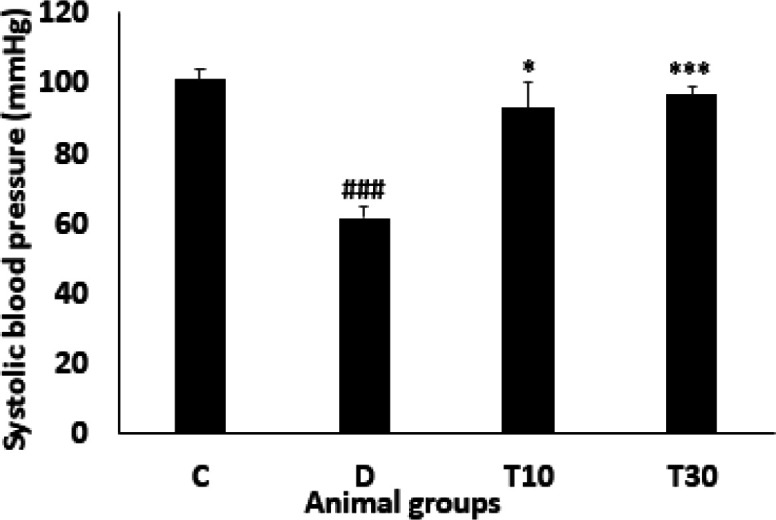
Systolic blood pressure (mean ± SEM, n = 8) in control (C), diabetic (D), diabetic treated with TMZ at 10 and 30 mg/kg (T10 and T30, respectively).^ ###^*P *< 0.001 *vs*. control group, ^*^P < 0.05, ^***^P < 0.001 *vs*. untreated diabetic group, one-way ANOVA followed by LSD *post hoc* test

The systolic blood pressure was significantly decreased in the diabetic animals compared with the control group (61.32 ± 3.4 mmHg vs. 101 ± 3 mmHg, *P *< 0.001). However, administration with TMZ at 10 and 30 mg/kg significantly inhibited this alteration compared with the untreated diabetic rats (93 ± 7.4 mmHg and 96.6 ± 2.29 mmHg vs. 61.32 ± 3.4 mmHg, *P *< 0.05 and *P *< 0.001, respectively, Figure 5).

## Discussion

This study was carried out to evaluate the beneficial effects of TMZ on vascular miRNAs, lipid profile, inflammation, and hypotension in the alloxan-induced diabetic animals. In line with previous studies, the present study also observed decreased miR-24, miR-126, blood pressure and increased inflammatory cytokines, oxidative stress and dyslipidemia in the diabetic rats. Furthermore, this study found that decreased TG, TC, inflammatory cytokines and oxidative stress with TMZ treatment significantly improved these alterations in vascular miRNAs in the diabetic rats. Collectively, these findings demonstrated an important role of vascular miRNAs in mediating vascular disorders in diabetes.

After administration of TMZ for eight weeks in the diabetic rats, blood glucose was improved in our investigation. One of the main mechanisms of TMZ treatment might be blood glucose improvement by reducing insulin-resistance. Researchers have also found that insulin-resistance reduction resulted from TMZ treatment is associated with the alleviated insulin signaling pathway phosphatidylinositol 3-kinase (PI3-K)/Akt (28). This pathway is an important factor in the insulin signal transduction and plays a key role in the metabolism of lipid and glucose. In addition, TMZ could increase translocation of GLUT4 to the sarcolemma in skeletal muscle cells (29). A previous study has demonstrated that activated AMP-activated protein kinase (AMPK) pathway is associated with TMZ administration. The AMPK pathway plays an important role in increasing uptake of glucose and fatty acids, glycolysis, as well as fatty acid oxidation (30). In addition, in diabetic rats treated with TMZ, increased insulin effect and glucose utilization have been demonstrated (31, 32).

In the present study, reduced body weight gain was observed in the diabetic rats. Body weight reduction may result from dehydration or metabolism impairment, or both (33). Therefore, decreased blood glucose may cause increased body weight. 

Hyperglycemia and dyslipidemia are associated with macrovascular complications and are linked to an enhanced risk of atherosclerosis (34, 35). 

Increased TC, TG, LDL-c, VLDL-c, and reduced HDL-c that contribute to atherosclerosis have been observed in the diabetic rats, which is consistent with a previous study (34). However, treatment with TMZ markedly improved the increase in TC, TG, and VLDL-c, suggesting the effect of TMZ treatment on dyslipidemia. On the other hand, reduction of free fatty acid levels in plasma has been demonstrated to cause TMZ treatment in diabetic rats (36). Thus, these findings showed an important role of TMZ treatment in reducing dyslipidemia induced by diabetes. Collectively, these results propose that improved blood glucose and lipid profile may act simultaneously to contribute to the improvement of body weight in the diabetic rats treated with TMZ. 

Oxidative stress and inflammation are associated with diabetes and are linked to atherosclerosis (37). In the present study, increased MDA level was found in the diabetic rats, which was improved by TMZ treatment. Similarly, decreased MDA level by TMZ administration has been indicated in animals subjected to myocardial ischemia-reperfusion (38). Inflammatory cytokines, including TNF-α and IL-6 are associated with diabetes and are linked to increased macro-vascular disorders. In the diabetic animals, we observed increased TNF-α and IL-6 levels, and these alterations were improved with TMZ administration, which is coherent with a previous study in patients with stable coronary artery disease. (1,39).

Consistent with previous investigations (12, 40), we demonstrated that diabetes significantly reduced plasma miR-24 and miR-126 levels compared to the control animals. However, the diabetic rats treated with TMZ significantly increased the levels of both microRNAs, proven that are effective regulators of vascular integrity and angiogenic signaling in the vascular system (41). A previous study has presented that decreased plasma miR-126 in type 2 diabetic patients may cause increase β cells injury in the pancreas and high blood glucose (42). Hyperglycemia increased ROS, TNF-α and IL-6, and reduced miR-24 and miR-126 levels in plasma. Decreasing miR-24 and miR-126 levels increased the ROS and inflammatory markers induced by diabetes. Both miRNAs negatively regulated ROS, IL-6, and TNF-α (43, 11). In addition, decreased miR-126 expression by inflammatory cytokines and increased miR-126 level by anti-inflammatory agents have been reported in endothelial cells and colonic myofibroblasts (44, 45). The decreased plasma miR-24 level that contributes to endothelial dysfunction has been observed in the diabetic rats, which is coherent with a previous study in diabetic mice (46). In summary, our results propose that increased plasma miR-24 and miR-126 levels may reduce oxidative stress and inflammation by TMZ treatment in the diabetic rats. Therefore, reducing oxidative stress and inflammation by TMZ treatment may play a key role in decreasing cardiovascular complications in diabetes. 

In the diabetic rats, we observed a decrease in blood pressure. The reason for the hypotension may result from reduced cardiac output, impaired cardiac autonomic and elevated plasma nitric oxide in the diabetic rats (47, 48). Nevertheless, TMZ administration significantly improved the hypotension in the diabetic rats, which may be attributed to the improvement of oxidant/anti-oxidant ratio and glucose metabolism and/or ventricular contractility that have been indicated to contribute to hypotension in diabetes (47,49). In addition, miR-24, by regulating VSMCs function, has likewise been reported to play an important role in vascular resistance (50). Therefore, miR-24 may be involved in decreased hypotension in the diabetic rats, treated with TMZ, and it may require further studies.

**Table 1:** Lipid profile (mean ± SEM, n = 8) in control, diabetic and diabetic administrated with TMZ at 10 and 30 mg/kg

**Groups**	**C**	**D**	**T10**	**T30**
TC (mg/dL) TG (mg/dL) HDL-c (mg/dL) LDL-c (mg/dL) VLDL-c (mg/dL)	46.22 ± 1.37 36.33 ± 5.52 35 ± 0.53 24.44 ± 0.5 7.26 ± 1.10	61.75 ± 2.32^##^ 52.87 ± 4.78^#^ 28.80 ± 2.39^#^ 26.75 ± 0.70 10.57 ± 0.94^#^	48.12 ± 2.43^**^ 24.62 ± 2.59^**^ 26.87 ± 1.63 25.1 ± 1.38 4.92 ± 0.51^**^	45.90 ± 3.93^***^ 30.10 ± 4.99^**^ 31 ± 0.71 24 ± 0.61 6.02 ± 0.99^**^

C, control; D, diabetic; T10 and T30 diabetic treated with TMZ at 10 and 30 mg/kg. TC, total cholesterol; TG, triglycerides; HDL-c, high-density lipoprotein cholesterol; LDL-c, low-density lipoprotein cholesterol; VLDL-c, very low-density lipoprotein cholesterol. ^#^ *P *< 0.05, ^##^ *P *< 0.01 *vs*. control group, ^** ^*P *< 0.01, ^*** ^*P *< 0.001 *vs*. untreated diabetic group, one-way ANOVA followed by LSD *post hoc* test.

## Conclusion

Taken together, our findings proposed that TMZ has protective effects on decreased plasma miR-24 and miR-126 levels, inflammation, dyslipidemia, and hypotension that may participate in endothelial dysfunction and atherosclerosis. 
